# 

**Published:** 1884-05

**Authors:** 


### CONTENTS:

ART. I.—Plastic Surgery of the Face. By Prof. L. McLane Tiffany, M. D. .......... 1
ART. II—A New Invention Useful to Dentists Working Continuous Gum and Plate Work. By D. Genese, D. D. S. .......... 9
ART. III—Phosphate of Lime and the Teeth. By C. M. Wright, D. D. S. .......... 10
ART. IV—.On the Influence of Micro Organism in Caries. By Arthur S. Underwood, M R. C. S., L. D. S. .......... 22
ART. V.—Salivary Calculus and its Removal. By Dr. J. F. Marriner .......... 32
Correspondence. By D. Genese, D. D. S. .......... 38
Change of Date .......... 39

### EDITORIAL, ETC.

The New Building of the Dental Department of the University of Maryland .......... 39
Maryland State Board of Dental Examiners .......... 40

### OBITUARY.

Dr. Samuel W. Neall .......... 41

### BIBLIOGRAPHICAL.

A System of Oral Surgery. By Prof. James E. Garretson, M. D., D. D. S. .......... 42

Gorgas' Dental Medicine .......... 43

Notes on Dental Practice. By Henry C. Qinby, D. D. S. .......... 44

Vulcanite and Celluloid. By L. Eldred Gilbert, D. D. S. .......... 45

Transaction of Ohio State Dental Society .......... 45

Transactions of Illinois State Dental Society .......... 45

### MONTHLY SUMMARY.

The Therapeutic Application of Nitrous Oxide Gas .......... 45
Artificial Crowns .......... 47
Ether .......... 47

